# The use of a pedicled buccal fat pad for reconstruction of posterior mandibular defects

**DOI:** 10.1186/s40902-021-00306-6

**Published:** 2021-07-06

**Authors:** Hyen Woo Lee, Sung ok Hong, Heeyeon Bae, Youngjin Shin, Yu-jin Jee

**Affiliations:** 1 Department of Oral and Maxillofacial Surgery, Kyung Hee University Dental Hospital at Gangdong, #892 Dongnam-ro, Gangdong-gu, Seoul, 05278 Republic of Korea; 2 Department of Oral and Maxillofacial Surgery, College of Dentistry, School of Dentistry, Kyung Hee University, Seoul, Republic of Korea

**Keywords:** Mandibular defect, Buccal fat pad, Reconstruction, Pedicled flap, Oral defect

## Abstract

**Background:**

The pedicled buccal fat pad has been used for a long time to reconstruct oral defects due to its ease of flap formation and few complications. Many cases related to reconstruction of defects in the maxilla, such as closing the oroantral fistula, have been reported, but cases related to the reconstruction of defects in the mandible are limited. Under adequate anterior traction, pedicled buccal fat pad can be a reliable and effective method for reconstruction of surgical defects in the posterior mandible.

**Case presentation:**

This study describes two cases of reconstruction of surgical oral defects in the posterior mandible, all of which were covered by a pedicled buccal fat pad. The size of the flap was sufficient to perfectly close the defect without any tension. Photographic and radiologic imaging showed successful closure of the defects and no problems were noted in the treated area.

**Conclusion:**

In conclusion, the pedicled buccal fat pad graft is a convenient and reliable method for the reconstruction of surgical defects on the posterior mandible.

## Background

After the first report of successful buccal fat pad (BFP) graft for closure of the oroantral and oronasal communications by Egyedi [1], literature reports have illustrated that the pedicled BFP can be grafted for closure of defects in various regions in the oral cavity. Most of the published literature reports tend to focus on reconstruction of the maxilla or buccal mucosa, due to the limited anterior traction of the BFP to the mandible. This study presents a series of 2 cases of posterior mandibular defect reconstruction with the pedicled BFP. The data were obtained by reviewing operative and medical records. The BFP was used to reconstruct large-sized surgical oral defects on the posterior mandible due to either radiation-induced osteonecrosis or tumor resection. Herein, authors also present a comprehensive literature review on application of the buccal fat pad graft on the posterior mandible. The purpose of this case report is to explain the feasibility and effectiveness of the reconstruction of the posterior mandible using pedicled BFP.

## Case presentation

### Case 1

**Fig. 1:**
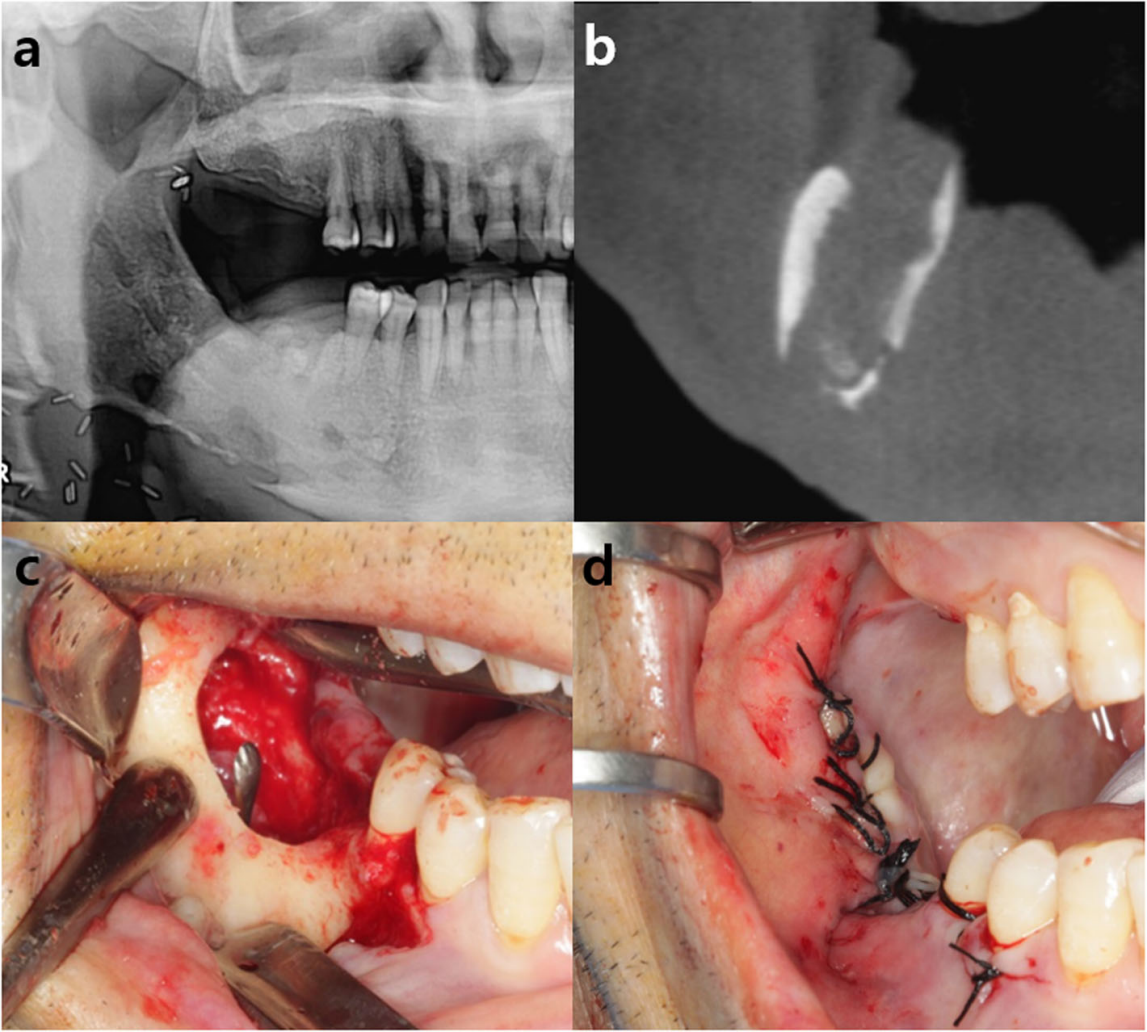
**a**, **b** Preoperative panoramic view and CT view showing partial osteonecrosis on the right mandibular body and angle. **c** Intra-oral photograph showing communicated fistulas on the intra- and extra-oral cavity after sequestrectomy via intra-oral approach. **d** The communicated fistulas on the intra- and extra-oral cavity were sutured for primary closure

**Fig. 2:**
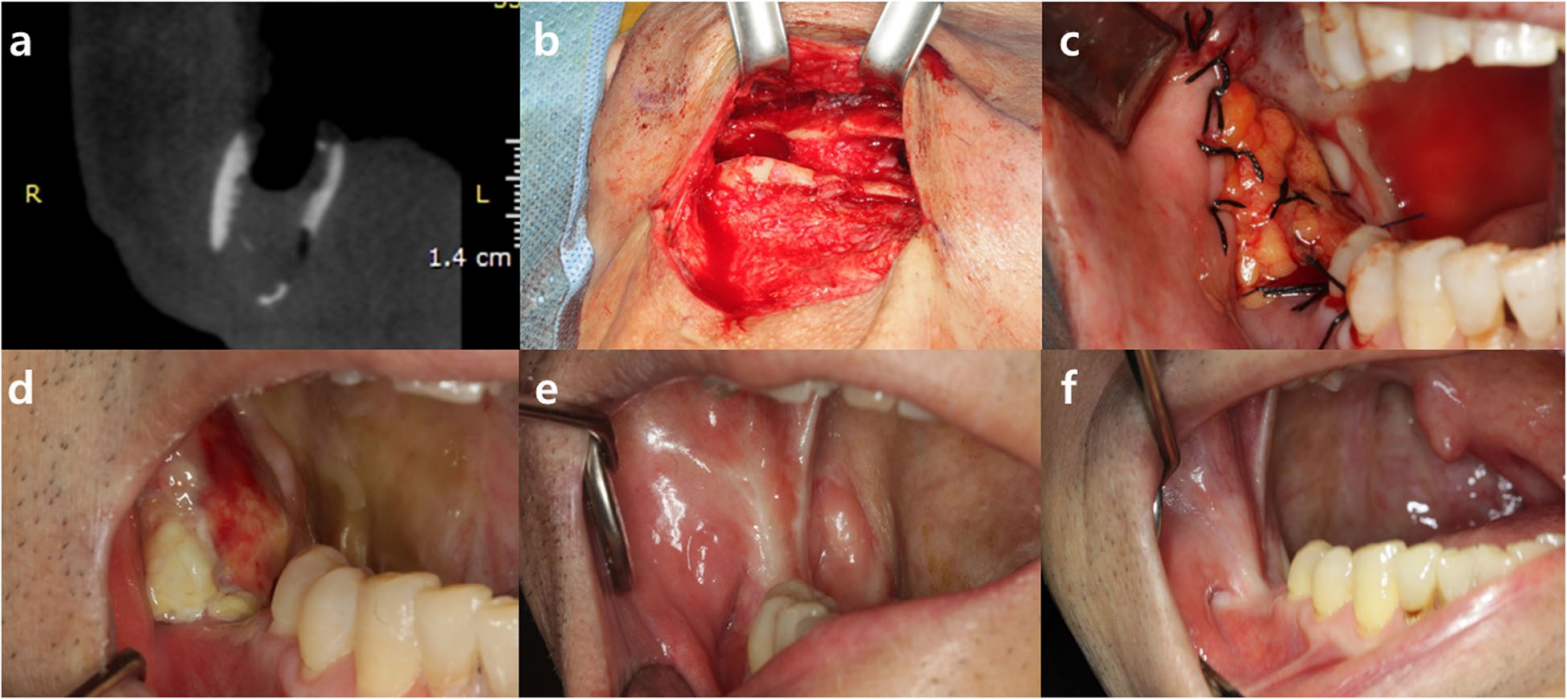
**a** CT view showing continuous state of osteonecrosis on the mandibular body and angle. **b** Large intra-oral surgical defect connecting the oral cavity and affected mandibular bone on the retromolar region was seen after the removal of inflamed tissue. **c** The BFP flap perfectly covered the intra-oral defect without tension, and flap was sutured to marginal oral mucosa with 3-0 black slik. **d**, **e**, **f** Re-epithelization of the grafted buccal fat pad after operation was seen (**d** 4-week, **e** 6-month, **f** 1-year follow-up)

A 69-year-old man presented with swelling of the right mandible and pus discharge from the ipsilateral posterior lower dentition accompanied with the tooth mobility. The patient had a clinical history of resection of the squamous cell carcinoma on the right tonsil with reconstruction and subsequent radiation therapy 4 years ago. After a series of clinical and radiological examinations, partial osteonecrosis on the right mandibular body and angle was shown (Fig. 1a-b). After extracting the teeth with poor prognosis, the patient underwent surgery for removal of the sequestra and inflammatory tissues via an intra-oral approach. After the resection, communicated fistulas on the intra- and extra-oral cavity were seen and sutured for primary closure (Fig. 1c-d). At 3 months of follow-up, continuous osteonecrosis of the mandibular body and angle was shown with pus discharge from both the intra- and extra-oral fistula (Fig. 2a). Accordingly, the patient underwent sequestrectomy via an extra-oral approach. After the removal of all inflamed tissue, a large intra-oral surgical defect on the retromolar region connecting the oral cavity and affected mandibular bone was seen (Fig. 2b). Ipsilateral pedicled BFP was used for reconstruction of the defect. After a 2-3 cm, mucosal incision was made at least 2 cm below the Stensen’s duct, the buccinator, and zygomaticus major were cut and blunt dissection was done for opening and herniation of the fat pad. The BFP was carefully harvested and pulled out to avoid injury of capsule. By gently pulling the flap anteriorly toward the defect without tension, the defect was perfectly covered with the flap. The flap margin was sutured to the marginal oral mucosa using 3-0 black slik for fixation (Fig. 2c). The extra-oral opening site was sutured with 3-0 vicryl and 5-0 nylon. The fistula in the oral cavity was closed 1 month after the pedicled BFP graft, and the condition was stably maintained without additional complications throughout 1-year follow-up period (Fig. 2d-f).

### Case 2

**Fig. 3:**
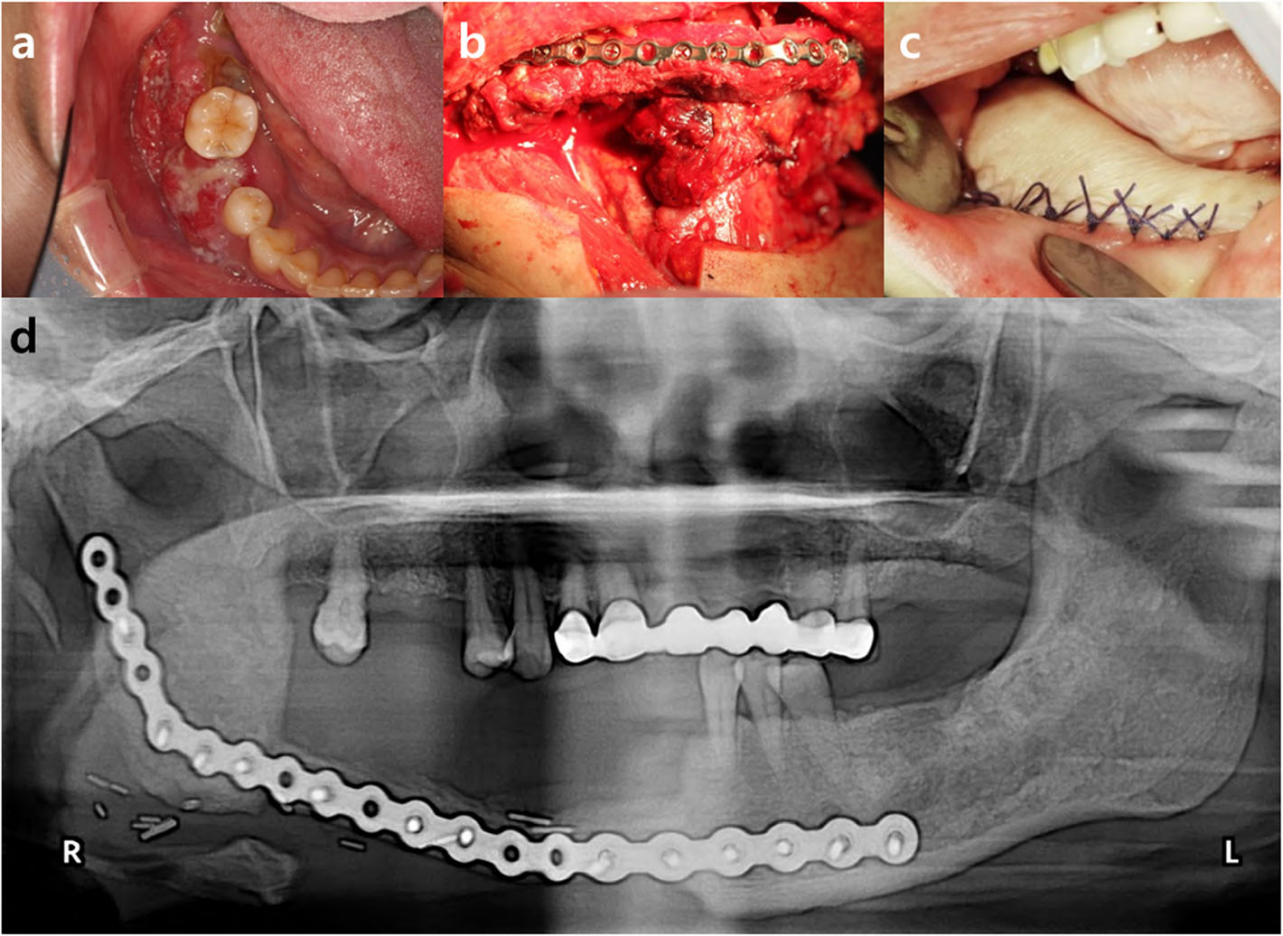
**a** The patient was diagnosed with squamous cell carcinoma in the right mandible. **b**, **c** The patient underwent right hemi-mandibulectomy, fibula-free flap reconstruction, and split-thickness skin graft with thigh. **d** Postoperative panoramic view showing the reconstruction with fibula-free flap and split-thickness skin graft after the right hemi-mandibulectomy

**Fig. 4:**
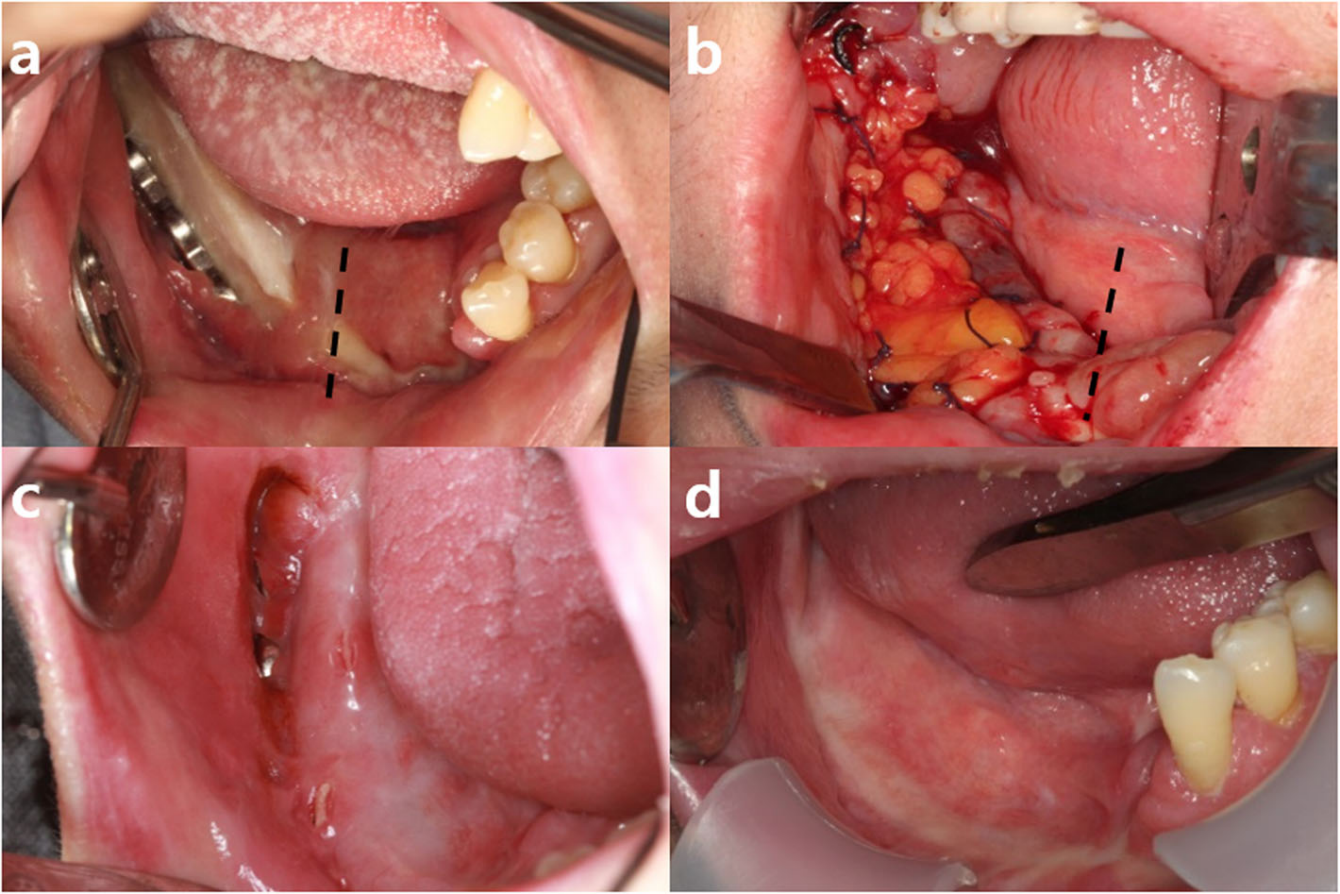
**a** Grafted fibula bone was maintained in good condition but partial exposure of reconstruction plate and grafted bone showed from the retromolar to the premolar region (midline marked with a dotted line). **b** The pedicled BFP flap perfectly covered the intra-oral defect without tension, and flap was sutured to marginal oral mucosa with 3-0 black slik (midline marked with a dotted line). **c** Part of the metal plate was still exposed after the pedicled BFP graft, but the condition remained stable. **d** Re-epithelization of the grafted buccal fat pad was complete after the removal of the reconstruction metal plate (5-month follow-up)

The patient was a 53-year-old female who was diagnosed with squamous cell carcinoma in the right mandible 2 months ago. She underwent right hemi-mandibulectomy, fibula-free flap reconstruction, and split-thickness skin graft from the thigh (Fig. 3). After 2 months of surgery, the grafted fibula bone was in good condition, but partial necrotic change of the reconstructed soft tissue was observed. After debridement of the necrotic and inflamed soft tissue, epithelization was seen but with exposure of the reconstruction plate and grafted fibula from the retromolar to the premolar region (Fig. 4a). The ipsilateral pedicled BFP was grafted to cover the defect as the method described in case1 (Fig. 4b). Part of the metal plate was still exposed after the pedicled BFP graft, but the condition of the healing gingiva remained stable (Fig. 4c). After 7 months, the reconstruction metal plate was removed. The fistula in the oral cavity was fully closed after 8 months, and the condition remained stable without additional complications throughout the 3-year follow-up period (Fig. 4d-e).

## Discussion

Since the first report of a successful BFP graft for closure of the oroantral and oronasal communications by Egyedi in 1977 [1], applications of the BFP in different indications and parts of the intra-oral defects have been introduced [2–5]. Literature reports have illustrated the use of BFP flap for reconstruction of intra-oral defects after tumor removal including malignant tumor [6–9]. Intra-oral surgical defects due to medication-related osteonecrosis of the jaw or osteoradionecrosis treatment have also been successfully reconstructed with BFP flap [10, 11]. Singh et al.’s review on the efficacy of BFP grafting, consisting of 509 cases, generally focused on closure of the oroantral communication and cleft palate [12]. Toshihiro et al. reported 23 cases of the BFP grafting to cover surgical defects of the palate, maxilla, upper gingiva, buccal mucosa, lower gingiva, oral floor, and temporomandibular joint region [13].

According to Singh et al., the ideal defect to be reconstructed with a BFP is the maxillary defects due to its anatomical situation [5]. The BFP is situated in the masticatory space between the buccinator muscle and masseter muscle, surrounded within a facial envelope [6]. The BFP is divided into three lobes: anterior, intermediate, and posterior, which are encapsulated by an independent membrane [14]. The principal blood supply of BFP derives from the buccal and deep temporal branches of maxillary artery, transverse facial branch of the superficial temporal artery and few branches of facial artery [15]. Loukas et al.’s study of BFP evaluation with CT and MRI, mean weight of the BFP was 9.3 g and mean volume was 9.6 ml [16].

**Table 1:** Summary application of the BFP on the posterior mandible in 12 studies sorted by year of publication

No.	Author(s) (year)	Number of patients	Average age (range)	Cause	Location	Defect size (mm)
1	Tideman et al. (1986) [15]	6	65 (54-75)	• Mucoepidermoid carcinoma • SCC	• Angle/ramus mandible (1) • Posterior mandible (5)	—
2	Baumann et al. (2000) [17]	2	—	• Unspecified	• Retromolar region	—
3	Hao et al. (2000) [18]	2	—	• Malignant tumor Unspecified	• Retromolar region	—
4	Rapidis et al. (2000) [4]	3	—	• Tumor unspecified	• Posterior mandible	50 × 50 × 10 70 × 50 × 20 50 × 40 × 20
5	Colella et al. (2004) [7]	3	54 (42-75)	• Verrucous carcinoma • Pleomorphic adenoma • SCC	• Retromolar region	(Max. diameter) 40 30 30
6	Chakrabarti et al. (2009) [6]	1	67	• Verrucous carcinoma	• Retromolar region	30 × 20
7	Toshihiro et al. (2013) [13]	2	61 (59-63)	• SCC	• Lower gingiva (molar region)	30 × 25 28 × 28
8	Ohba et al. (2013) [9]	1	70	• SCC	• Lower gingiva (retromolar region)	—
9	Rotaru et al. (2015) [11]	7	77 (72-81)	• Medication-related osteonecrosis	• Posterior mandible (6) • Ascending ramus to the contralateral mandibular incisor (1)	— 62 × 18
10	Habib et al. (2016) [2]	1	54	• SCC	• Retromolar region	40×36
11	Zhang et al. (2017) [19]	2	47.5 (39-56)	• SCC	• Retromolar region	—
12	Present cases (2021)	2	61 (53-69)	• Radiation-induced osteonecrosis • SCC	• Retromolar region (1) • Retromolar to premolar region (1)	—

As seen in previously published literature reports, BFP graft cases related to mandibular defect reconstructions are somewhat limited due to its anatomical situation. Although there have been several reports illustrating mandibular defect reconstruction using BFP, most of the reports were confined to the retromolar trigone area. To investigate and evaluated the reported cases reconstructed with BFP in the posterior mandible, a comprehensive literature review was performed from 1986 onwards and were compared in a table to provide data on average age, location, defect size, and cause of the reconstruction (Table 1). A PubMed search of the terms “buccal fat pad graft” and “buccal fat pad reconstruction” was performed from 1977 to 2021. The focus of the review was to investigate and evaluate the cases reconstructed with BFP in the posterior mandible; hence, cases involving buccal mucosa reconstruction or temporomandibular joint reconstruction were excluded. Studies involving free BFP graft or combination with another local or pedicled flap were also excluded from the present review for clear evaluation. Overall, 12 papers and a total of 32 patients, including the present cases were chosen for the review based on the criteria for the study.

The median age of the patients, limited to the patients for whom the information was provided, were 64.7 (range, 39–81 years). Of 32 cases, defects were induced by malignant tumor (*n* = 19), medication-related osteonecrosis (*n* = 7), unspecified tumor (*n* = 3), unspecified cause (*n* = 2), and radiation-induced osteonecrosis (*n* = 1). It was found that BFP has been used most commonly for reconstruction of defect in retromolar region. Recently published studies show that the range of BFP application to the anterior part of mandible has been expanded. Location of the reconstructed mandibular defect site ranged posteriorly from angle/ramus and anteriorly to the contralateral mandibular incisor. Size of the defect ranged from minimum of 30 × 20 mm to maximum of 70 × 50 × 20 mm.

In previous series of case reports from other authors, BFP was able to reach the maxilla as far anteriorly as the canine tooth and slightly beyond the midline of the palate [2] while some were not able to reach the midline of the palate [5]. Posteriorly, the tuberosity, soft palate, and retromolar area were all easily reached by the BFP [2]. In this study, however, the authors showed that the pedicled BFP can be successfully applied on the posterior mandible area that reaches up to the premolar region beyond the retromolar trigone in case 2. Despite a single case, Rotaru et al.’s report shows a successful reconstruction of mandibular defect ranging from ascending ramus to the contralateral mandibular incisor. This case report supports our opinion on feasibility of BFP to cover ipsilateral mandibular premolar region without complications. However, further research is still needed to investigate the range of reconstruction on the mandible using BFP.

There is a limitation in the size of the mandibular defect which could be reconstructed with the BFP graft. Posterior mandibular defect size up to 70 × 50 × 20 mm can be successfully reconstructed with the BFP, in which the BFP is placed over a rich vascular bed provided by the musculature of the recipient area [4].

Spontaneous epithelization of BFP flap occurs within 4-6 weeks which makes no need for an additional skin grafting when used intra-orally [15]. Pedicled BFP flap has numerous advantages over other flaps on reconstruction of surgical oral defects: (1) very acceptable to patient, (2) high success rate due to its rich blood supply, (3) good healing with minimal scar and morbidity, and (4) versatile usage with other flaps and materials. On the other hand, local mucosal flap has the limitation when covering large oral defects because it often fails due to the poor vascularized network of the recipient bed [20]. The microvascular flap can be used to cover large oral defects owing to its rich bloody supply but it also has the disadvantage of morbidity of the donor site.

Several drawbacks of the BFP when reconstructing oral defects exist. The BFP has a size limitation, can be used only once and can have postoperative complications such infection, fistula opening, or mouth opening limitation. Colella et al. reported limited mouth opening in five of 15 cases reconstructed using the BFP flap, which was due to scar retraction and the loss of separation of the muscles of mastication from each other. Physical therapy for 4 to 6 weeks after the surgery was recommended in such cases [7]. To minimize the incidence of postoperative complications, Tideman at al. suggested covering and suturing the surgical defect without tension and have the patient to receive a liquid or soft, non-chewy diet until the soft tissues completely healed [15]. It is also advised to calculate the individual volume of BFP from radiographic images such as computed tomography (CT) or magnetic resonance imaging (MRI) to assess the possible amount of BFP for oral defect reconstruction [2, 8, 13].

## Conclusions

In the present cases, two large post-operative mandibular defects were successfully closed with BFP grafts. Although BFP has not been widely used in mandibular defect reconstruction due to limitation of anterior traction, if adequately used, it can be a convenient and reliable method for the reconstruction of surgical defects on the posterior mandible.

## Data Availability

Not applicable.

## Abbreviations

BFP: Buccal fat pad
CT: Computed tomography
MRI: Magnetic resonance imaging
